# LDLR Expression and Localization Are Altered in Mouse and Human Cell Culture Models of Alzheimer's Disease

**DOI:** 10.1371/journal.pone.0008556

**Published:** 2010-01-01

**Authors:** Jose F. Abisambra, Tina Fiorelli, Jaya Padmanabhan, Peter Neame, Inge Wefes, Huntington Potter

**Affiliations:** 1 Byrd Alzheimer's Institute, Tampa, Florida, United States of America; 2 Department of Molecular Medicine, University of South Florida, Tampa, Florida, United States of America; 3 Eric Pfeiffer Suncoast Alzheimer's and Gerontology Center, Tampa, Florida, United States of America; 4 Florida Alzheimer's Disease Research Center (NIA), Tampa, Florida, United States of America; New York State Institute for Basic Research, United States of America

## Abstract

**Background:**

Alzheimer's disease (AD) is a chronic neurodegenerative disorder and the most common form of dementia. The major molecular risk factor for late-onset AD is expression of the ε-4 allele of apolipoprotein E (apoE), the major cholesterol transporter in the brain. The low-density lipoprotein receptor (LDLR) has the highest affinity for apoE and plays an important role in brain cholesterol metabolism.

**Methodology/Principal Findings:**

Using RT-PCR and western blotting techniques we found that over-expression of APP caused increases in both LDLR mRNA and protein levels in APP transfected H4 neuroglioma cells compared to H4 controls. Furthermore, immunohistochemical experiments showed aberrant localization of LDLR in H4-APP neuroglioma cells, Aβ-treated primary neurons, and in the PSAPP transgenic mouse model of AD. Finally, immunofluorescent staining of LDLR and of γ- and α-tubulin showed a change in LDLR localization preferentially away from the plasma membrane that was paralleled by and likely the result of a disruption of the microtubule-organizing center and associated microtubule network.

**Conclusions/Significance:**

These data suggest that increased APP expression and Aβ exposure alters microtubule function, leading to reduced transport of LDLR to the plasma membrane. Consequent deleterious effects on apoE uptake and function will have implications for AD pathogenesis and/or progression.

## Introduction

Alzheimer's disease (AD) is a chronic neurodegenerative disorder and the most common form of dementia. Currently, almost 50% of the population over 85 years of age suffers from AD. Onset of the disease after age 65 is described as late-onset or sporadic AD, which accounts for over 95% of the cases and has an idiopathic etiology. Extracellular β-amyloid deposits in the cores of neuronal (senile) plaques and in vessel walls, intraneuronal neurofibrillary tangles, and neuroinflammation characterize the disease's pathology resulting in accelerated neuron loss and dementia [1]. Amyloid deposits are the result of abnormal processing of the amyloid precursor protein (APP) by two enzymes: β- and γ-secretase. Mutations in the two presenilin (PS) genes encoding the catalytic core of γ-secretase as well as mutations in the APP gene lead to increases or alterations in Aβ, a 38–42 amino acid peptide and the seed for, and major component of amyloid pathology. The particular structure of Aβ_42_, which is the most pathogenic form, confers the ability to self-aggregate, oligomerize, and, dependent on the presence apolipoprotein E (apoE), to polymerize into amyloid filaments [2]–[4].

The ε-4 isoform of apoE is the strongest molecular risk factor for the development of AD. About 60–80% of AD patients have at least one copy of apoE4 [2], [4], [5] and the risk for AD is increased in an ε-4 allele dose-dependent manner [6]. ApoE is a 34 kDa, 299-amino acid glycoprotein and is the chief cholesterol transporter in the central nervous system (CNS). It's gene, located on chromosome 19q13, may code for any homozygote or heterozygote combination of three common isoforms, apoE2, apoE3, and apoE4 [7], [8]. In the CNS, apoE-cholesterol is principally made in astrocytes and exported to neurons [9], [10]; however, neurons can also produce apoE-cholesterol during stress [11]. Despite the presence of several receptors that are capable of internalizing apoE such as low-density lipoprotein receptor (LDLR), LDLR-related protein (LRP), apoER2, and VLDLR, in neurons apoE is mostly imported via the LDLR [12]–[15].

LDLR is a membrane-spanning glycoprotein that plays a critical role in removing LDL and VLDL from the blood [16], [17]. Under low intracellular sterol levels, LDLR gene expression is primarily and directly activated by sterol response element-binding proteins (SREBPs) [18] and secondarily by thyroid hormone [19]. The translation of LDLR mRNA yields a 120 kDa protein that is post-translationally modified in the Golgi apparatus into the mature, 160 kDa LDLR [20], [21]. The mature receptor can be divided into five regions: the N-terminal ligand-binding domain [17], [22], the epidermal growth factor precursor homology domain [22], [23], the *O*-linked polysaccharide domain [24] where the protein is post-translationally modified, the membrane-spanning domain [25]–[27], and the C-terminal cytoplasmic domain [25]. Upon maturation, LDLR is transported to the cell membrane via a clathrin-coated pit vesicle [28]. On the membrane, the ligand-binding domain is exposed extracellularly to associate and internalize LDL or VLDL, mediated by apoB or apoE, respectively. Once inside the cell, LDLR-ligand-containing vesicles are acidified by proton pumps [29], leading to uncoupling of the receptor-ligand complex. At this point, the LDL or VLDL-cholesterol undergoes further processing to be readily available for the cell's requirements.

Several groups identified a potential contribution of LDLR to AD and investigated potential links, for example by crossing AD transgenic mice with the LDLR−/− mouse model of hypercholesterolemia to investigate the effects of LDLR deficiency [30]–[40]. Some apparently opposing results were obtained. Here we investigate the effects of APP over-expression on the expression and localization of LDLR to identify possible changes that could result in an altered apoE metabolism. We report that over-expression of APP in a human neuroglioma cell line increased the amounts of LDLR mRNA and protein, and the receptor accumulated in the perinuclear region of the APP-expressing cells. This altered localization was specific to LDLR and could not be seen for LRP. Furthermore, in comparing NTG with PSAPP and APP−/− transgenic mouse models, we found that LDLR protein levels were directly proportional to the amount of APP. Immunohistochemical analysis of α- and γ-tubulin suggest that alterations in LDLR are rooted in an APP-mediated disturbance of the centrosome and microtubules, preventing proper transport of LDLR to the plasma membrane.

## Results

**Figure 1:**
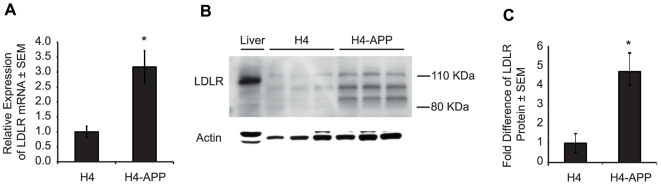
LDLR mRNA and protein are upregulated in H4-APP cells compared to H4 controls. (A) RT-PCR quantification of LDLR mRNA expression level in H4 and H4-APP cells (n = 3; *p = 0.04). LDLR threshold cycle values were normalized to GAPDH. (B) Western blot for LDLR in whole-cell lysates from H4 and H4-APP cells. Lane 1 contains liver whole-cell lysate from a NTG mouse. (C) Quantification of western blot (n = 3; *p = 0.01). All bands for LDLR were quantified and their values shown in this graph. The LDLR band densities were normalized to the actin band in each lane. RT-PCR and western blot experiments were conducted in triplicate.

Initially, we sought to determine changes in the mRNA and protein levels of LDLR in human neuroglioma cells that were stably transfected with human wild-type APP (H4-APP) and used their non-transfected H4 counterpart as controls. H4-APP cells express about 12 times more APP than non-transfected controls and it is distributed throughout the whole cell (Figure S1). RT-PCR experiments with H4 and H4-APP cells demonstrated a 3-fold increase in LDLR mRNA in H4-APP cells compared to H4 controls (Figure 1A). This result was paralleled at the protein level as evidenced by western blot analysis (Figure 1B). Quantification of the immunoblots showed a 4-fold increase in LDLR protein in H4-APP cells compared to H4 controls (Figure 1C).

**Figure 2:**
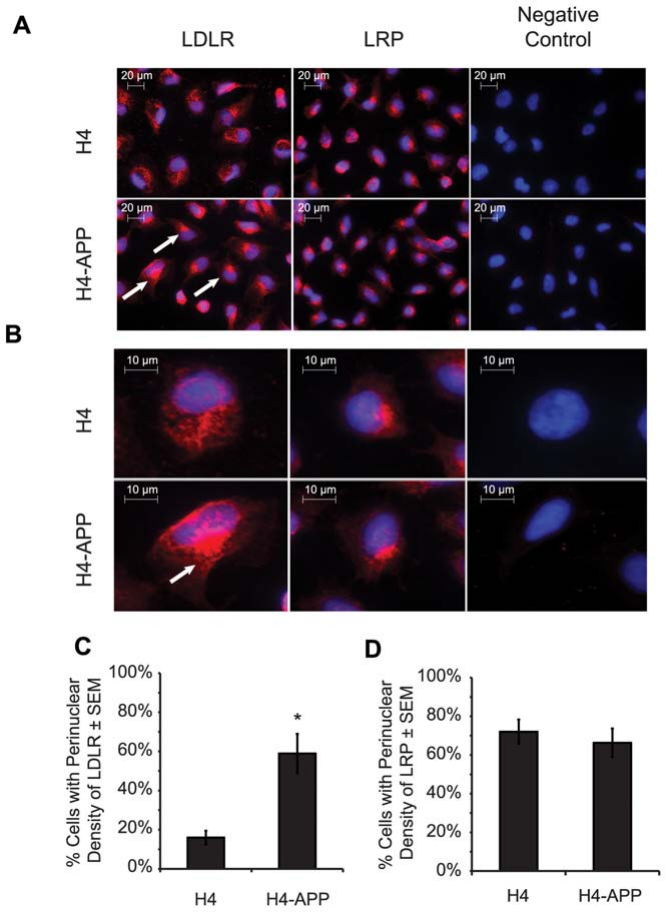
LDLR distribution is altered in H4-APP cells compared to H4 controls. (A) Immunohistochemistry imaging at 400× magnification of LDLR and LRP in H4 and H4-APP cells. Red signal corresponds to LDLR or LRP as indicated and blue signal corresponds to Hoechst-labeled cell nuclei. Arrowheads point to three examples of a dense perinuclear LDLR-positive signal present in H4-APP cells. (B) Larger image of a selected cell from panel 2A; the arrowhead points to an LDLR-positive density. (C and D) Quantification of the percentage of cells with perinuclear density of fluorescent signal in LDLR (C) and LRP (D), which is described in more detail in Figure S2.

Next, we performed immunofluorescence imaging on H4 and H4-APP cells to determine changes in LDLR localization. In order to assess whether potential APP-induced alterations were specific to LDLR, we immunostained for LDLR and another member of the LDLR family, LRP (LDLR-related protein-1) (Figure 2A). Images of H4 and H4-APP cells without primary antibody incubation are shown as a negative control of the assay (Figure 2A). While we did not observe significant changes in the distribution of LRP, we found that in H4-APP cells, LDLR had become densely concentrated in the perinuclear region (indicated by the arrowheads in panels 2A and 2B). Compared to LDLR localization, the LRP signal was concentrated in a perinuclear density in both H4 and H4-APP cells. At closer inspection (Figure 2B), we confirmed that in H4 control cells, LDLR is homogeneously distributed, whereas in H4-APP cells, LDLR appears to converge to form a dense perinuclear core. To quantify this effect, we defined cells displaying a dense LDLR-positive focus as cells containing a signal that was three standard deviations above the background and with at least 1000 pixels^2^. By this criterion, 59% of H4-APP versus 10% of H4 cells had LDLR accumulated in the perinuclear zone of the cells. (Figure 2C; *p = 0.001). In contrast, 72% of H4-APP cells and 66% of H4 cells displayed the dense perinuclear signal for LRP, not indicating any significant change (Figure 2D; *p* = 0.56).

**Figure 3:**
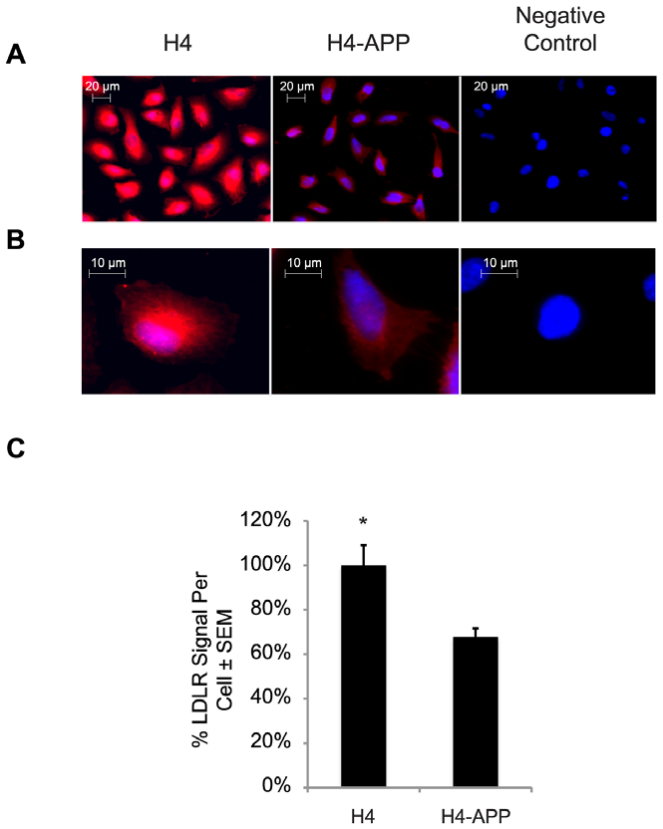
Cell surface LDLR is much reduced in H4-APP cells. H4 and H4-APP cells were immunostained for LDLR in the absence of detergents to prevent permeabilization of the plasma membrane and allow antibody access only to the cell surface. (A) Images magnified at 400× showing LDLR signal (red) and nuclei (blue) for H4 and H4-APP. (B) Zoomed image of one cell isolated from panel A. (C) Quantification of the average LDLR-positive signal per cell showing a 32% reduction of LDLR on the membrane of H4-APP cells (*p = 0.03). The average of the ratio of the total LDLR intensity and the number of cell nuclei for the H4 condition was equaled to 100%. The H4-APP ratio was divided by the H4 ratio of LDLR intensity per cell; A total of ∼18,000 cells were taken into account from three independent experiments.

It seems reasonable that if LDLR becomes highly concentrated in a single perinuclear core, then there may be a relative deficit of LDLR in its normal, physiologically relevant location on the plasma membrane. We therefore examined H4 and H4-APP cells with the LDLR antibody as above, but leaving out the permeabilization step so as to only visualize cell-surface LDLR. H4-APP cells exhibited a 32% significant reduction of plasma membrane-associated LDLR (Figure 3A, 3B, and 3C). This result indicated that there was likely a concomitant reduction in LDLR function, despite the compensatory upregulation of LDLR mRNA and protein shown in Figure 1.

**Figure 4:**
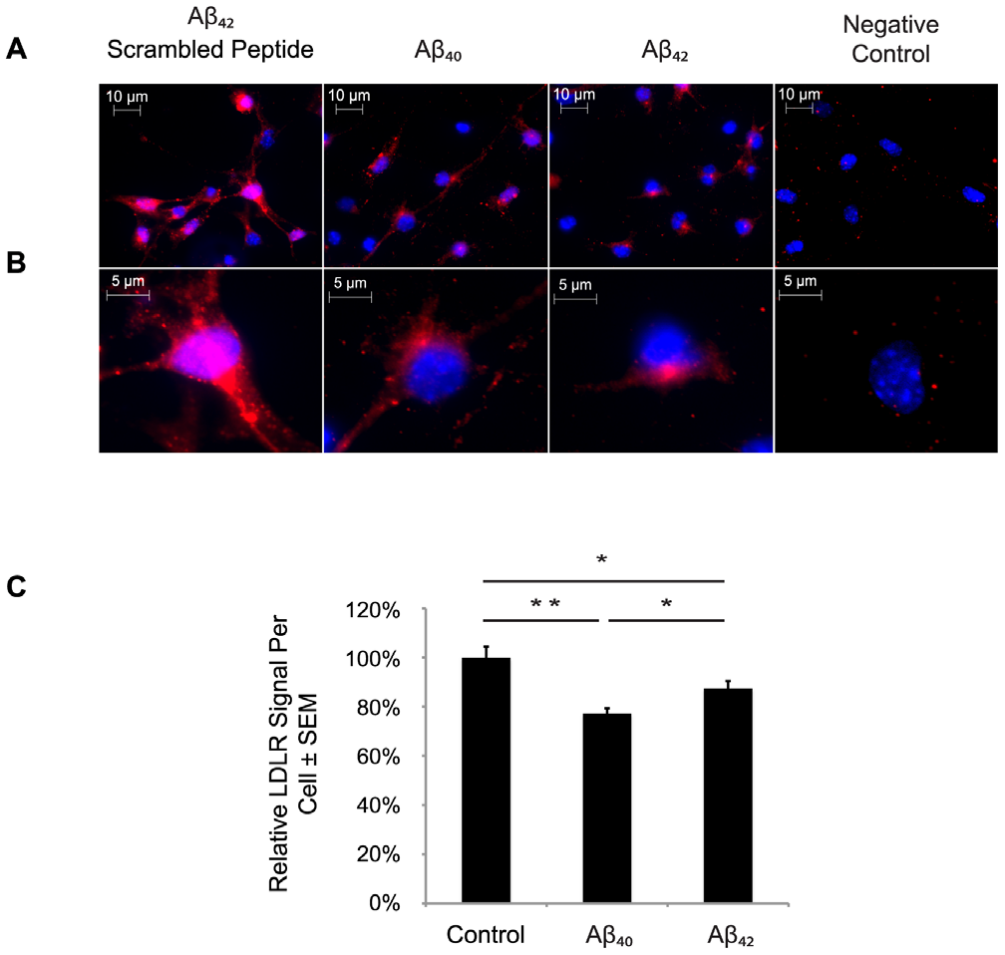
Aβ_42_ reduces LDLR cell surface localization in primary neurons of NTG mice. Primary neurons were obtained from E18 fetuses, plated and grown for one week, and treated with 1 µM Aβ_40_ or Aβ_42_ for 48 hours. Cell surface LDLR was immunostained (red). (A) Image of primary neurons from cells treated for 48 hours with 1µM Aβ_42_ scrambled peptide, Aβ_40_, and Aβ_42_; image is magnified 630×. (B) Zoomed image isolating one cell in each field of panel A. The negative control corresponds to staining in the absence of primary antibody. Quantification as in Figure 3 revealed statistically significant reduction in cell surface LDLR induced by exposure to Aβ.

AD pathology is initiated and maintained when the APP protein becomes proteolytically cleaved to generate various forms of the Aβ peptide, the 1–42 amino acid version being one of the most pathogenic. In order to assess the general relevance of the redistribution of the LDLR in H4-APP cells, we repeated the experiment using cultured mouse cortical neurons from normal mice and exposing them to 1uM of either Aβ_40_ or Aβ_42_ for 48 hours; as a reference, we also treated and immunostained a set of neurons with a peptide consisting of scrambled amino acids of the Aβ_42_ peptide (Figure 4A and 4B). Similar to the H4-APP cells, the Aβ_40_- and Aβ_42_-treated cortical neurons had 23% (*p<0.05) and 13% (**p<0.01) less surface LDLR compared to Aβ_42_ scrambled peptide-treated cells (Figure 3C). The amount of surface LDLR was also significantly decreased in Aβ_40_-treated compared to Aβ_42_-treated cells (10%; *p<0.05).

**Figure 5:**
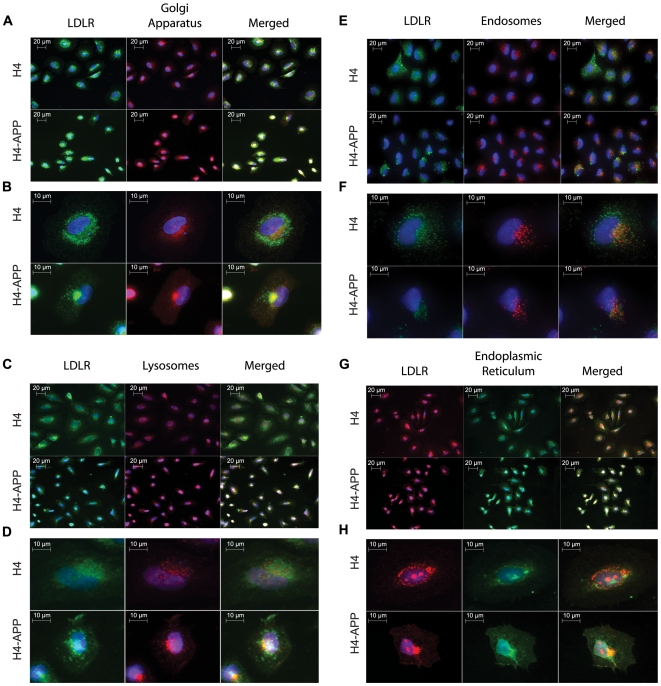
LDLR is abundant in the *trans*-Golgi network of H4-APP cells. H4 and H4-APP cells were co-stained for LDLR and different organelle markers. A and B show Golgi apparatus at low and high magnification, respectively. C and D show LDLR co-stained with lysosomal marker at low and high magnification, respectively. E and F show LDLR co-stained for endosomes at low and high magnification respectively. G and H show LDLR co-stained with endoplasmic reticulum at low and high magnification, respectively. A, C, E, and G were taken at 400×, while B, D, F, and H are zoomed images.

Thus far we have shown that the upregulated LDLR protein in H4-APP cells cannot be accounted for on their cell membrane. To identify the localization of the LDLR aggregate, we co-stained LDLR with organelle markers for the Golgi apparatus (Figure 5A and 5B), lysosomes (Figure 5C and 5D), endoplasmic reticulum (Figure 5E and 5F), and early endosomes (Figure 5G and 5H). We found that in H4-APP cells, LDLR signal co-localized in the Golgi apparatus and lysosomes, or the *trans*-Golgi network. In contrast, LDLR signal in the ER or endosomes was not particular to either H4 or H4-APP cells.

**Figure 6:**
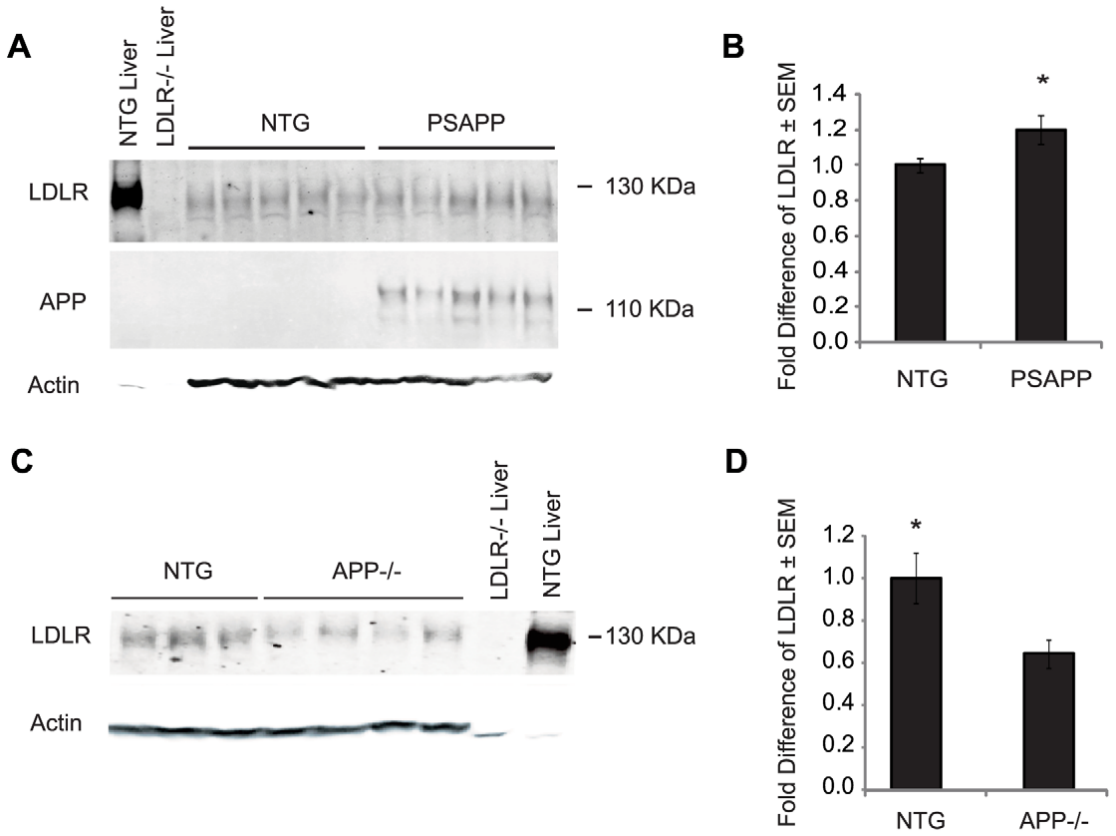
Brain LDLR is increased in PSAPP mice and decreased in APP−/− mice compared to controls. Whole-cell lysates were prepared from brains of 10-month old PSAPP, APP−/−, and age-matched non-transgenic control mice for western blot analyses; liver whole cell lysates were prepared from NTG and LDLR−/− as positive and negative control homogenates, respectively. (A) Western blot for LDLR, APP, and actin from PSAPP and NTG control lysates. (B) Quantification of LDLR signal normalized to actin in western blot of panel A (n = 5; *p = 0.05). (C) Western blot for LDLR and actin from NTG and APP−/− mice. (D) Quantification of LDLR signal normalized to actin in western blot of panel C (n_NTG_ = 3 and n_APP−/−_ = 4; *p = 0.04).

We next sought to reproduce the APP-induced LDLR over-expression and mis-localization in a physiologically relevant experimental system—an *in vivo* model of AD. We chose PS1+/−APP+/− (PSAPP) mice at 10 months of age when their brains are burdened with amyloid, homogenized brain tissue from the PSAPP mice and age-matched non-transgenic (NTG) controls and performed western blot analysis for LDLR. We observed a modest, yet significant increase of LDLR in PSAPP mice compared to controls (Figure 6A and 6B; 20% increase with *p = 0.05). The same experiment served to confirm that the PSAPP mice overexpressed APP (Figure 6A). In order to further investigate whether the LDLR protein levels are influenced by APP expression, we performed western blots for LDLR in 10-month old APP−/− mice. Interestingly, we found that LDLR was decreased by 55% in these mice compared to age-matched controls (Figure 6C and 6D; *p = 0.04). These experiments indicate that LDLR expression directly correlates with APP expression.

**Figure 7:**
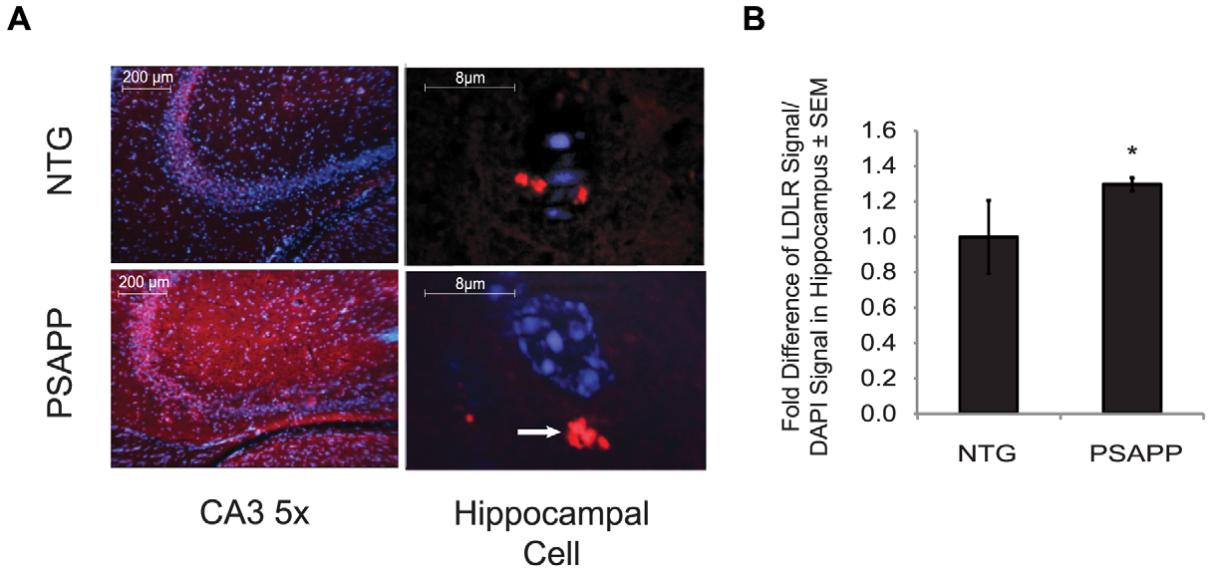
LDLR is increased and delocalized in the hippocampus of PSAPP mice compared to NTG controls. (A) Representative images at 5× magnification of immunohistochemistry staining of the CA3 region of the hippocampus and an enlarged view of a representative hippocampal cell surrounding the neuronal layer of a PSAPP and NTG mouse. Mice were 10-month old PSAPP and NTG. LDLR signal is in red and cell nuclei are in blue. Arrowhead in PSAPP hippocampal neuron indicates the concentration LDLR-positive signal. (B) Quantification of LDLR-positive signal normalized by the DAPI signal in hippocampus of PSAPP and NTG mice. Experiments were done in triplicate using brain sections of 8 mice for each condition.

To determine if changes in LDLR expression are paralleled by changes in its localization in PSAPP mice, we performed immunohistochemistry on brain tissue sections of PSAPP mice of 10 months and age-matched controls. We detected an increase in the LDLR signal in the hippocampus of PSAPP mice, a region that is particularly affected by the amyloid pathology. This effect was the strongest in the CA3 region of the hippocampus (Figure 7A). At higher magnification, we found that cells surrounding the neuronal layer of the hippocampus in PSAPP tissues also showed a dense accumulation of LDLR similar to that observed in H4-APP cells (arrowhead in PSAPP hippocampal cell of figure 7A), whereas the NTG counterpart lacked that same signal concentration (Figure 7A). After quantification of the LDLR signal normalized to the amount of DAPI signal in each field, we calculated a 30% increase (*p = 0.04) in LDLR in the hippocampi of PSAPP mice compared to NTG controls (Figure 7B).

**Figure 8:**
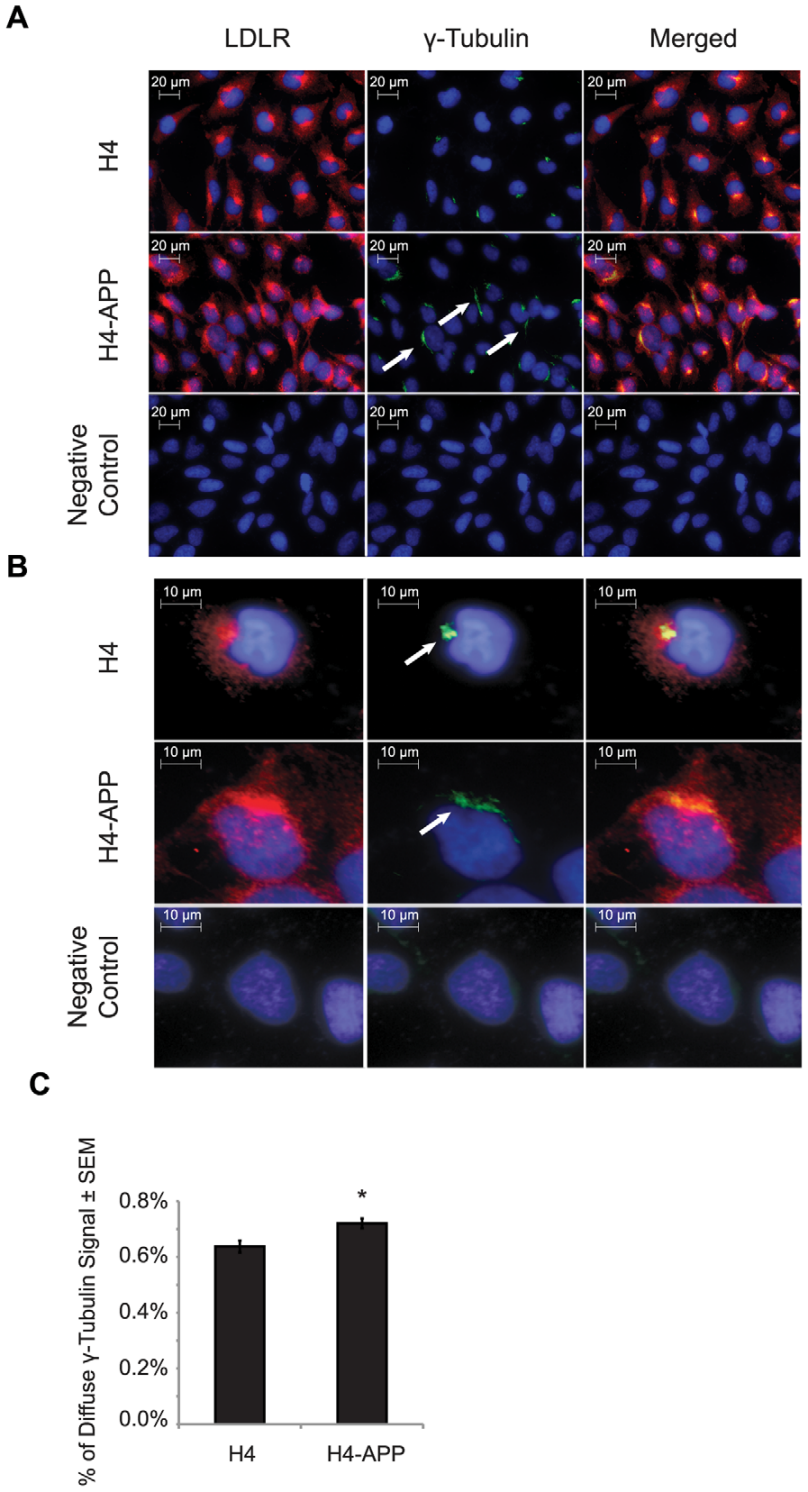
γ-tubulin signal is more widely distributed in H4-APP cells compared to H4 controls. (A) Immunohistochemistry imaging at 400× magnification of LDLR and γ-tubulin in H4 and H4-APP cells. Red, green, and blue signals correspond to LDLR, γ-tubulin, and cell nuclei, respectively. Arrowheads indicate three examples of H4-APP cells containing greater area of γ-tubulin signal distribution. (B) Larger image of a selected cell from the same slide. Merged images in A and B show the location of LDLR in relation to γ-tubulin and the nucleus. (C) Percentage of diffuse γ-tubulin signal in H4 compared to H4-APP cells. Quantification is described in further detail in Figure S2.

The centrosome, or microtubule-organizing center (MTOC), is responsible for the nucleation step preceding the polymerization of microtubules and maintains the structure of the microtubule network. It was previously reported that PS1 and APP bind to the centrosome [41], [42]. We therefore reasoned that the mechanism behind the changes in localization and expression levels of LDLR mRNA and protein could be based on an APP-mediated alteration of the microtubule-trafficking system. To address this hypothesis, we performed immunohistochemistry in H4 and H4-APP cells targeting γ-tubulin as a marker of the MTOC. While we observed condensed staining of γ-tubulin in H4 cells, H4-APP cells showed a diffuse, non-nuclear pattern of γ-tubulin signal (Figure 8A and 8B) where 0.64% of the γ-tubulin signal was dispersed in H4-APP cell. In contrast, 0.72% percent of the γ-tubulin signal was diffuse in the H4 cells; this was a modest change (0.08%), yet it was significantly different (*p = 0.01; Figure 8C).

**Figure 9:**
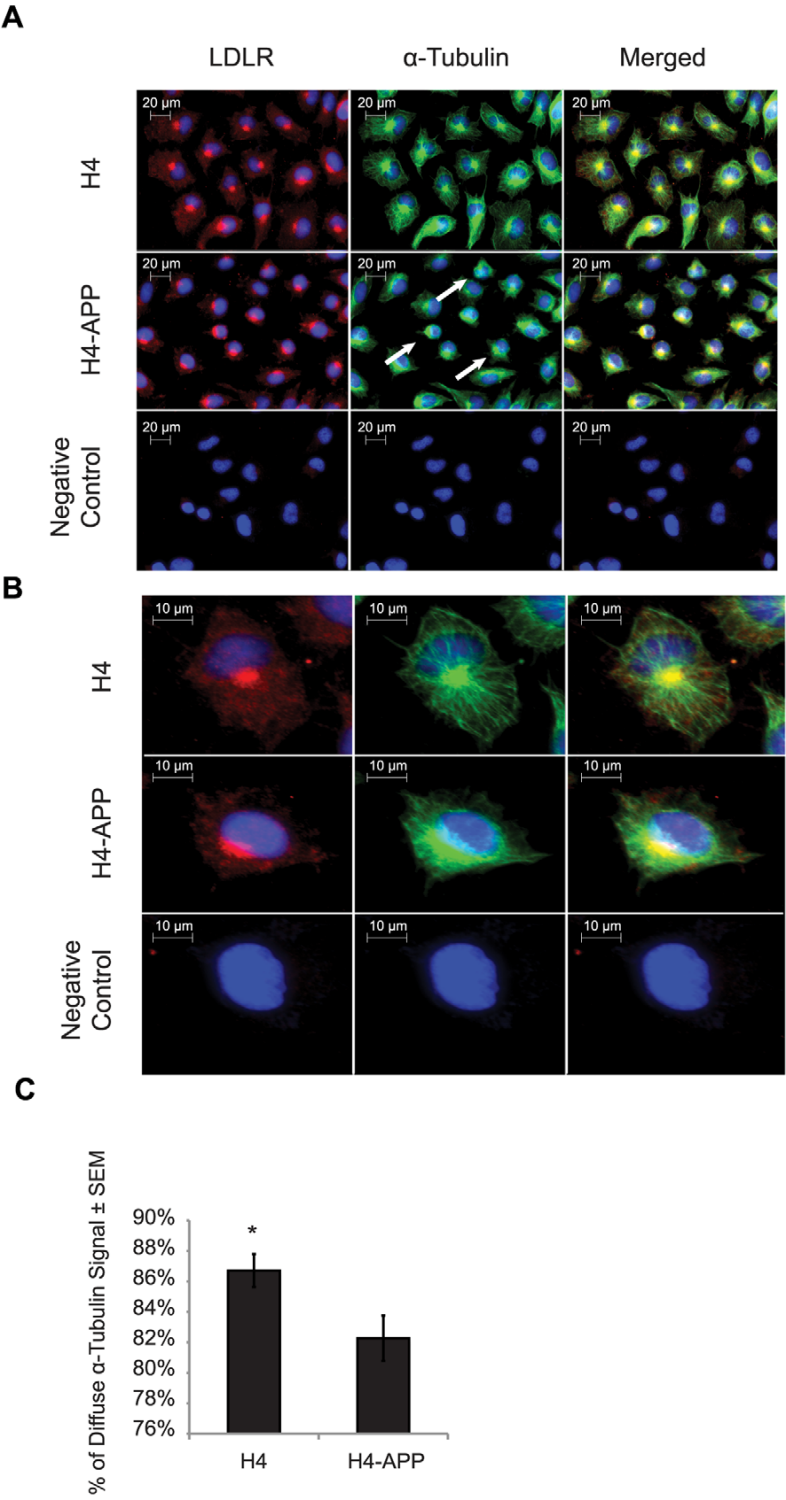
α-tubulin is less widely distributed in H4-APP cells compared to H4 controls. (A) Immunohistochemistry imaging at 400× magnification of LDLR and α-tubulin in H4 and H4-APP cells. Red, green, and blue signals correspond to LDLR, α-tubulin, and cell nuclei, respectively. Arrowheads indicate three examples of H4-APP cells containing less diffuse α-tubulin. (B) Larger images of selected cells from panel A. Merged images in A and B show the location of LDLR in relation to α-tubulin and the nucleus. (C) Percentage of diffuse α-tubulin signal in H4 compared to H4-APP cells. Quantification is described in further detail in Figure S2.

Finally, to address the relationship between APP over-expression and alterations to the mature microtubule-trafficking network, we performed immunofluorescent staining of H4 and H4-APP cells targeting α-tubulin as an indicator of microtubule localization. We found that only 82% of the alpha-tubulin signal in H4-APP cells was diffuse, while H4 cells had 87% of the total α-tubulin signal spread throughout the cell (Figure 9A and 9B). Like in the γ-tubulin staining experiment, this was a modest but significant change in signal distribution (5%; *p = 0.04) between the transgenic and non-transgenic (Figure 9C).

## Discussion

**Figure 10:**
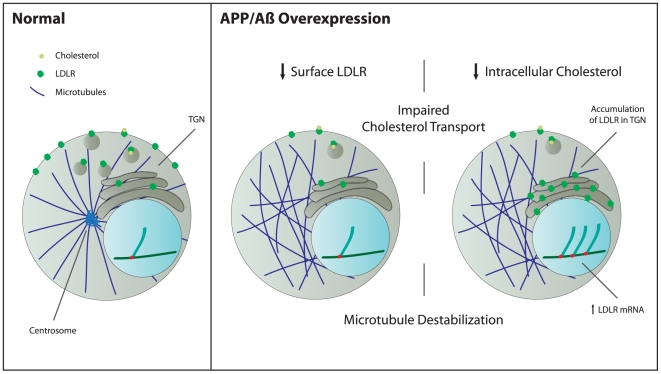
Proposed mechanism by which APP/Aβ overexpression diminishes LDLR trafficking by disrupting microtubule formation. Overexpression of APP/Aβ causes microtubule destabilization by altering the centrosome, and consequently, polymerized microtubules. As a result, LDLR trafficking from the TGN to the plasma membrane is impaired. Therefore, LDLR accumulates in the TGN. The implications are that the cell may be unable to import cholesterol effectively, which causes transcriptional activation of the LDLR gene.

Characterization of apoE metabolism in the brain is of critical importance for development of potential therapeutic targets for AD. Because apoE is the principal ligand for LDLR, alterations to LDLR trafficking are likely to impact AD pathology as well. As the main cholesterol transporter in the CNS, apoE is produced by glia and delivers cholesterol cargo to neurons by receptor-mediated endocytosis via LDLR. In order for apoE-cholesterol to enter the cell, LDLR must be localized to the plasma membrane. Our findings indicate that in H4-APP cells and primary neurons treated with Aβ_42_, this localization is altered in that the majority of LDLR signal is concentrated in a dense focus in the perinuclear zone and is therefore hindered from reaching the plasma membrane (Figures 2 and 3). Transcriptional activation of the LDLR gene is normally induced by a system that is sensitive to low levels of intracellular sterols [18]. Therefore, it is possible that the upregulation of LDLR (Figure 1A) in H4-APP cells is a reflection of low intracellular cholesterol due to the significant reduction of LDLR on the cell membrane for cholesterol internalization (Figures 3, 4). A summary diagram indicating the observed changes in LDLR production and localization induced by APP/Aβ is shown in Figure 10.

Dietary and endogenous biosynthesis of cholesterol modulates the rate at which APP is processed into Aβ. Studies using animals fed high cholesterol diets revealed an increase in amyloid plaque formation in rabbits and in transgenic mouse models of AD [43], [44], [45]; moreover, in these mice, the high cholesterol diet induced cognitive decline [46]. *In vitro* studies show that imbalances in cellular cholesterol may favor APP cleavage by either α- or γ-secretase. Conversely, cholesterol-depleted rat hippocampal primary neurons show reduced APP processing into Aβ [47], [48] and favor the generation of non-amyloidogenic APP processing by α-secretase to yield the soluble, non-pathogenic protein sAPPα [49], [50]. In contrast, γ-secretase processing is favored when APP is located in the lipid raft; the result is formation of toxic Aβ peptide. Thus, the amount and distribution of cellular cholesterol is essential for the formation of lipid rafts, and therefore the localization and processing of APP. This connection might also mean that if the observed increased expression of LDLR in apparent compensation for incorrect localization induced by APP overshoots, then more apoE-cholesterol could be imported, leading to more APP processing to Aβ in an accelerating pathogenic cycle.

Our experiments on PSAPP mice show that LDLR and APP protein levels are directly proportional (Figure 6). These results together with those obtained with the H4 and H4-APP cell models suggest that APP over-expression affects the MTOC such that LDLR transport to the cell membrane is significantly abrogated. Consequently, net cholesterol import into the cell could be decreased, leading to upregulation of LDLR transcription and protein levels. It was previously shown that APP and PS1 bind to the centrosome [41], [42]. As a result, the centrosome's nucleation function for microtubule formation may be disrupted by association with excess levels of APP or Aβ in our over-expression models or by mutations of the APP gene. This in turn may have widespread detrimental effects for the entire microtubule trafficking system. Our results provide evidence that trafficking of other proteins, organelles, or vesicles in the cell may also be disturbed, although this cannot be true for all proteins as LRP localization was unchanged in the H4-APP cells. (Figure 2A).

Over the past 15 years, a considerable amount of effort has been dedicated to characterize the potential participation of LDLR in AD pathology. This interest is based on data procured from linkage analyses of AD risk and the LDLR gene, preliminary data from population and case-control studies, and the importance of LDLR function in the regulation of cholesterol homeostasis via apoE metabolism, whose e4 allele is the most important risk factor for ‘sporadic’ AD besides age. Linkage analyses were based on the fact that the LDLR gene locus and a region associated with high frequency of AD risk share a common location on chromosome 19 [34], [35]. Furthermore, this peak of risk for AD onset is independent of the risk imparted by the nearby apoE4 gene [35]. Consequent attempts to characterize linkage of LDLR and AD onset yielded at least ten, case-control association studies and one family-based study [30]–[33], [36]–[40], [51], [52]. Of particular interest were polymorphisms contained in exons 8, 10, 13, and 15, as they had been proposed to have associations with risk of AD onset [33], [37], [39]. The overall conclusion of these studies is, however, still developing.

Other conflicting reports have investigated LDLR's participation in AD at the molecular level [46], [53], [54]. The objective of these studies was to study whether elimination of LDLR expression would affect the pathology in AD mice. To this end Fryer *et. al.* [53] and Cao *et. al.* [54] crossed the LDLR−/− mouse with mouse models of AD: the PDAPP and Tg2576, respectively. Both groups agreed that LDLR is the main regulator of apoE in CNS as they observed a significant increase in apoE levels in the CNS of their mice. However, Fryer *et. al.* did not observe changes in pathology, whereas Cao *et. al.* reported a mild yet significant increase in plaque deposition. Furthermore, this latter study also reported that the mice performed poorly on a battery of cognitive tests. The question of whether LDLR is involved in AD pathogenesis and/or progression remains unanswered.

In contrast to the investigations of the role of LDLR in AD, our approach was to determine instead the effects of amyloid pathology on LDLR metabolism. We describe in this report that APP/Aβ over-expression in *in vivo* and *in vitro* models of AD causes alterations to LDLR that may be explained by an APP/Aβ-mediated effect on the microtubule trafficking system. In H4-APP cells, a human neuroglioma cell line stably over-expressing human wild-type APP, LDLR mRNA and protein levels are increased. In these conditions, H4-APP had LDLR aggregated in the *trans*-Golgi network, which precluded its trafficking to the cell membrane. Furthermore, Aβ_42_ treatment also caused aggregation of the LDLR signal. Concomitantly, the PSAPP mouse model of AD, which over-expresses the full-length human APP containing the V717F mutation, also shows aberrant distribution and a mild, yet significant increase in LDLR protein. The experiments suggest that a likely explanation for these phenomena is that H4-APP cells have changes in the localization of the microtubule proteins α- and γ-tubulin.

The H4-APP cells and PSAPP mouse models we evaluated emulate the APP-induced amyloidogenic effects seen in individuals with trisomy 21. Interestingly, the serum cholesterol and lipid profiles of these individuals are abnormal, yet they are protected against atherosclerosis [55], [56]. It would be interesting to assess whether LDLR turnover rates are changed in different tissues of individuals with trisomy 21.

Based on our data, we suggest that, rather than a precursor, DS and AD dyslipidemia is a consequence of AD-like Aβ and amyloid production induced by increased APP. Indeed Aβ production and amyloid production has been shown to reduce cell surface levels of other proteins including the NMDA receptor and the EphB2 receptor, both involved in synaptic plasticity [57], [58]. Careful evaluation of the mechanisms underlying APP processing as a factor in cholesterol metabolism and receptor localization may yield novel therapeutic approaches against Alzheimer's, for example through rescuing general receptor transport to the cell membrane by reducing the deleterious effect of Aβ on the cytoskeleton.

## Materials and Methods

### Ethics Statement

All animal studies were approved by the University of South Florida's Institutional Animal Care and Use Committee and abided by that Committee's Policies on Animal Care and Use in accordance with the Guide for the Care and Use of Laboratory Animals, the Animal Welfare Regulations Title 9 Code of Federal Regulations Subchapter A, “Animal Welfare”, Parts 1–3, and the Public Health Service Policy on Humane Care and Use of Laboratory Animals. This USF program and the facilities for animal care and use are fully accredited by the Association for Assessment and Accreditation of Laboratory Animal Care International. The animals are in standard housing on a 12-hour light dark cycle and have food and water ad. lib.

### Materials

Quantitative-PCR experiments were performed using Applied Biosystems, PCR master mixes, human GAPDH endogenous control assays, and gene expression assays for human LDLR (assay ID Hs01092525_m1). Tissue culture reagents and electrophoresis supplies were purchased from Gibco/Invitrogen. Protein concentrations were determined with BCA™ (Pierce) colorimetric assays. Aβ peptides were obtained from American Peptide.

### Antibodies

Rabbit anti-LDLR antiserum was a generous gift from Dr. Joachim Herz at the University of Texas Southwestern. It was used as primary antibody for immunoblots (1∶1000) and immunohistochemistry assays (1∶100). [59]. Alternatively, a monoclonal anti-LDLR antibody from Fitzgerald Industries International (cat.# 10-L55A) was used for immunohistochemistry experiments where the co-stain targets required rabbit-polyclonal antibodies. Organelle markers for the Golgi apparatus, lysosomes, and early endosomes (GM130, LAMP1, and EEA1 antibodies, respectively) were purchased from Cell Signaling Technologies, while the KDEL antibody, a marker of endoplasmic reticulum was purchased from Stressgen. Monoclonal mouse anti-actin (Sigma-Aldrich) and AlexaFluor 488 and 594 (Invitrogen/Molecular Probes) antibodies were diluted according to the manufacturer for western blot (WB) and immunohistochemistry (IHC) assays, respectively. Goat anti-mouse IRDye®800CW and goat anti-rabbit IRDye®680 were purchased from LI-COR Biosciences (WB: 1∶15,000; IHC: 1∶1500). Monoclonal anti-α-tubulin and γ-tubulin antibodies were obtained from Sigma-Aldrich and diluted according to the company's specifications. Anti-LRP antibody was a generous gift from Dr. Guojun Bu.

### Animals

Mice with the genotype APP^+/−^, PS1^+/−^ were generated by crossing heterozygous PDGF-hAPP(V717F) mice [Swiss-Webster X C57BL/6] with PDGF-hPS1(M146L) heterozygotes [Swiss-Webster X C57BL/6] as described [60]. Non-transgenic (NTG) control mice for LDLR−/− mice were C57BL/6J expressing endogenous LDLR (The Jackson Laboratory). NTG control mice for APP^+/−^, PS1^+/−^ were littermates that lacked both transgenes. All mice were genotyped by PCR to confirm the presence or absence of PDGF-hAPP [61] and PDGF-hPS1 [62]. APP knock out mice (strain B6.129S7-*APP^tm1Dbo^*/J) were obtained from The Jackson Laboratory and were genotyped according to the provider's recommendations. Primary neurons were obtained from the cortex and hippocampus of E18 NTG mice as described [63]. Neurons were grown for one week on poly-L-lysine-coated 8-chamber slides in neurobasal medium with B27 supplement. Neurons were then treated for 48 hours with 1 µM concentration of either Aβ_40_ or Aβ_42_. Cells were then fixed and stained as described below in the immunohistochemistry section.

### Tissue Preparation

Brain tissue was acquired by anesthetizing mice with 0.1 mg/g Nembutal followed by transcardial perfusion with 0.9% saline solution for 8–12 min at 120 mmHg. Whole brains were immediately removed for processing. Messenger RNA was extracted by homogenizing tissues in TRI reagent (Sigma-Aldrich). Microsomal protein extracts were obtained as previously described [64], with minor modifications: 0.25M sucrose was prepared with protease inhibitors (1 tablet mini-Complete/10 ml sucrose, Roche Applied Science). Samples were dounce homogenized and spun at 10,000×g for 10 min. Then, supernatants were spun once at 30,000×g for 90 min in a fixed-angle rotor. Microsome pellets were resuspended in PBS with 10% glycerol and protease inhibitor cocktail (Roche). Brains for immunohistochemistry assays were fixed for 24 hrs in 4% *para*-formaldehyde. The fixed tissues were cryo-protected in successive sucrose gradients as previously described [65]. Brains were frozen on a temperature-controlled freezing stage, coronally sectioned (25 µm) on a sliding microtome, and stored in a solution of PBS containing 0.02% NaN_3_ at 4°C.

### Quantitative RT-PCR

Five micrograms of DNase-treated mRNA were reverse-transcribed with SuperScript® VILO™ cDNA Synthesis Kit (Invitrogen) using random hexamers according to the manufacturer's instructions. Quantitative RT-PCR was performed according to the manufacturer. Reactions were processed in the 7500 FAST System with its Sequence Detection Software (SDS) from Applied Biosystems.

### Western Blots

Unless otherwise indicated, 50 µg (protein) of brain microsomes were denatured with LDS sample buffer according to the Invitrogen protocol. Samples were loaded onto 3–8%, 1.0 or 1.5mm Tris-Acetate gels, and run at 80V for 180 min. Gels were dry- (iBlot, Invitrogen) or wet-transferred onto PVDF membranes for 9 min. Non-specific protein binding to the membrane was blocked by incubating with 5% BSA or 7% non-fat dry milk for 90 min at room temperature. Primary antibody for actin was diluted 1∶10000, while α-, γ-tubulin, and LDLR antibodies were diluted 1∶1000 in blocking buffer and incubated overnight at 4°C. Secondary antibody incubations with either IR-dyes (1∶15000) or HRP-conjugated were performed sequentially for 60 min at room temperature. Membranes were washed three times for 10 min with PBS or TBS and 0.1% Tween-20 after incubation with each antibody. Membranes were scanned and analyzed with the LI-COR Odyssey and accompanying software or developed using ECL reagent.

### Immunohistochemistry

H4 and H4-APP cells were cultured in 8-chamber slides for 3 days prior to immunostaining. Cells were then fixed in ice-cold methanol for 15 min at room temperature and incubated with blocking buffer (described below). Brain sections were mounted onto Colorfrost®/*Plus* slides (Fisher Scientific) Non-specific binding was blocked in NGS (10% normal goat serum, 0.2% Triton X-100, and 0.02% NaN_3_ in Tris-buffered saline (TBS)) or in NGS without detergents for non-permeabilizing experiments, for 120 min at room temperature. Primary antibodies were incubated overnight at 4°C in 10% NGS. After four, 5 min washes in TBS, slides were incubated with AlexaFluor −594 and −488 (anti-rabbit and anti-mouse, respectively) secondary antibodies in 10% NGS for 60 min at room temperature and washed in TBS. Slides were stained with Hoechst (1µg/ml in PBS) for 2 min to reveal cellular nuclei and mounted using GelMount (Fisher Scientific). Staining was analyzed with the Zeiss AxioImager.Z1 and AxioVision software using 5×/0.16 or 40×/0.75 dry ECPlan-NeoFluar objectives where specified. Images were captured at room temperature with an AxioCam MR3 camera. Fluorochromes used were DAPI, DsRed, and FITC. The magnified hippocampal cell image in figure 7A was modified in Adobe Photoshop by modifying the brightness and contrast to both the NTG and PSAPP cell images equally and simultaneously.

### Image Quantification

LDLR, LRP, γ and α-tubulins, and APP were quantified using ImageJ as described in Figure S2. Figure 3 and 4 analysis was performed by the following procedure: images scanning the entire well for H4, H4-APP, primary neurons, and no-primary-antibody negative controls were obtained. These images were taken at 100× and using the same exposure time for the channel corresponding to LDLR. We discarded pictures containing artifacts (such as those with cells damaged by the pipette tip), images from the edges of the wells (due to potential artifacts caused by the rubber gasket), and fields containing less than 200 nuclei. We obtained the integrated density (I.D.) of each field and the number of cell nuclei using ImageJ [66], [67]. We divided the I.D. by the number of cell nuclei to generate the amount of plasma membrane LDLR/cell (I.D./cell). The I.D./cell for the negative control was subtracted from the I.D./cell of H4, H4-APP, or neuronal cells. The average of the values for each condition was compared. Over 18,000 cell nuclei were taken into account from three independent experiments.

### Statistical Analyses

All data reported were obtained from independent experiments repeated at least three times. Data were plotted as ±SE of the mean. *P*-values were obtained from paired t-test analyses.

## Supporting Information

Figure S1 APP overexpression in H4-APP cells causes aberrant localization in the cell. (A) Immunohistochemistry imaging at 400× magnification of LDLR and APP in H4 and H4-APP cells. Red, green, and blue signals correspond to LDLR, APP, and cell nuclei, respectively. (B) Quantification graph of diffuse APP signal between H4 and H4-APP cells. (C) Western blot of APP in H4 vs. H4-APP cells; actin was used as a loading control. (D) Quantification of western blot in (C); quantification is described in more detail in Figure S2.
(1.41 MB TIF)

Figure S2 Quantification of staining using Image J. A) Representative image of H4-APP cells for analysis. The cells have been stained for LDLR. B) Histogram generated from ImageJ based on the image shown in panel A. Pixel gray value is the color of the pixel on a scale of 0 to 255, with 0 being absolute black and 255 being absolute white. C) Magnification of the histogram in panel B. In this representative histogram, the mean pixel value is 11.801, and the standard deviation of the histogram is 23.217. In order to identify the intensely stained perinuclear density, the image was thresholded at 3 standard deviations from the mean. At this threshold, only pixels with a gray value of 81 or greater were identified as positive stain (dark gray region in right tail). Identification of total cellular staining was done with the image thresholded at 0.5 standard deviations from the mean of the image, so all pixels with a value of 21 or greater were identified as positive stain (light gray region and dark gray region). For tubulin staining, staining outside of the density was defined as that falling between 0.5 standard deviations and 3 standard deviations (light gray region only). D) Image showing thresholding of the image shown in panel A at 3 standard deviations. Note that only intense staining is identified at this threshold. E) Further identification of the intense perinuclear density based on the criterion that the region be at least 1000 square pixels in area. F–K) Representative image of H4 cells stained for LDLR, thresholded, and perinuclear densities identified as described in A–E.
(0.56 MB TIF)
