# Supplementary figures and images for: LDLR Expression and Localization Are Altered in Mouse and Human Cell Culture Models of Alzheimer's Disease
Source: PLoS One. 2010 Jan 1;5(1):e8556. doi: 10.1371/journal.pone.0008556 (PMC2797391; doi:10.1371/journal.pone.0008556)

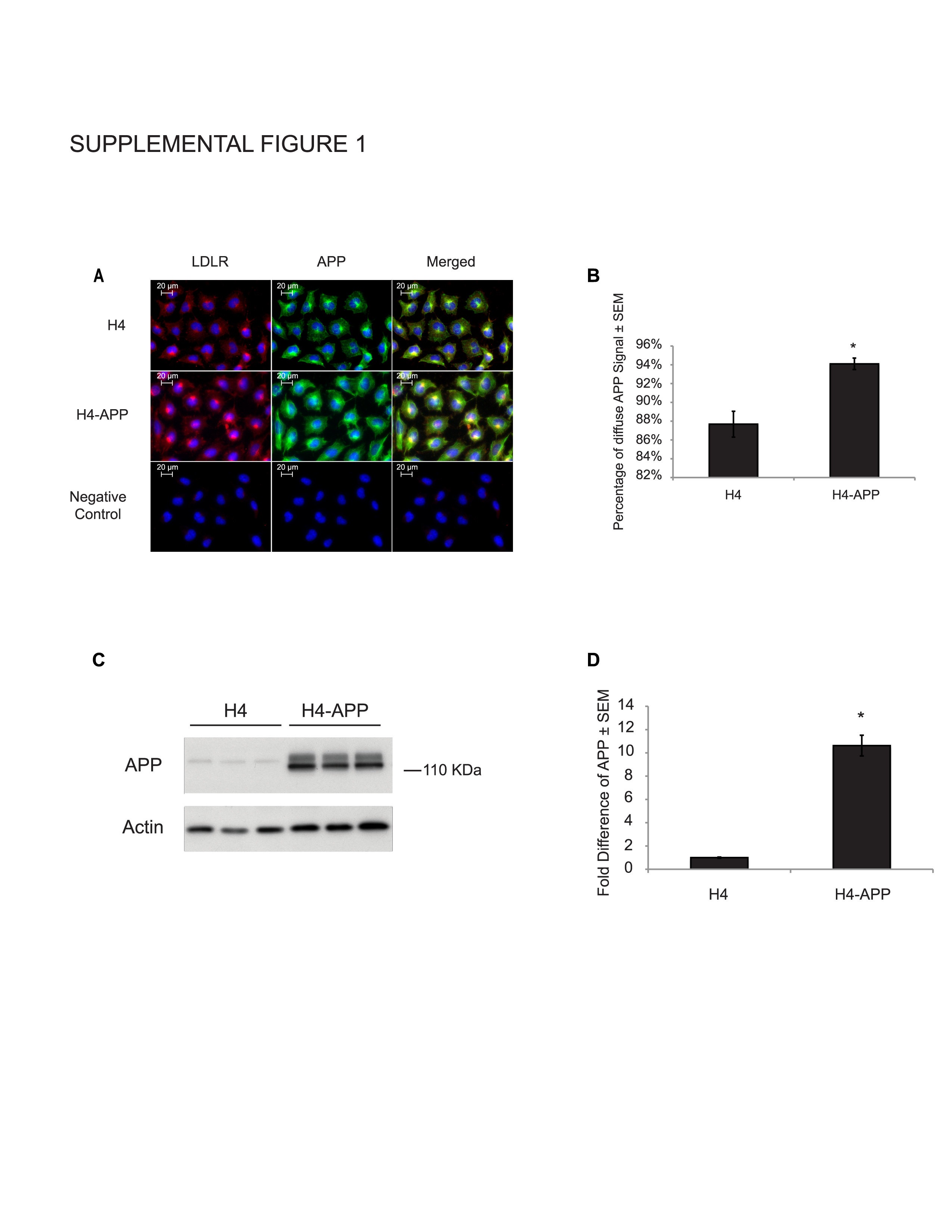

Supplement: Figure S1 — APP overexpression in H4-APP cells causes aberrant localization in the cell. (A) Immunohistochemistry imaging at 400× magnification of LDLR and APP in H4 and H4-APP cells. Red, green, and blue signals correspond to LDLR, APP, and cell nuclei, respectively. (B) Quantification graph of diffuse APP signal between H4 and H4-APP cells. (C) Western blot of APP in H4 vs. H4-APP cells; actin was used as a loading control. (D) Quantification of western blot in (C); quantification is described in more detail in Figure S2. (1.41 MB TIF) [file pone.0008556.s001.tif]

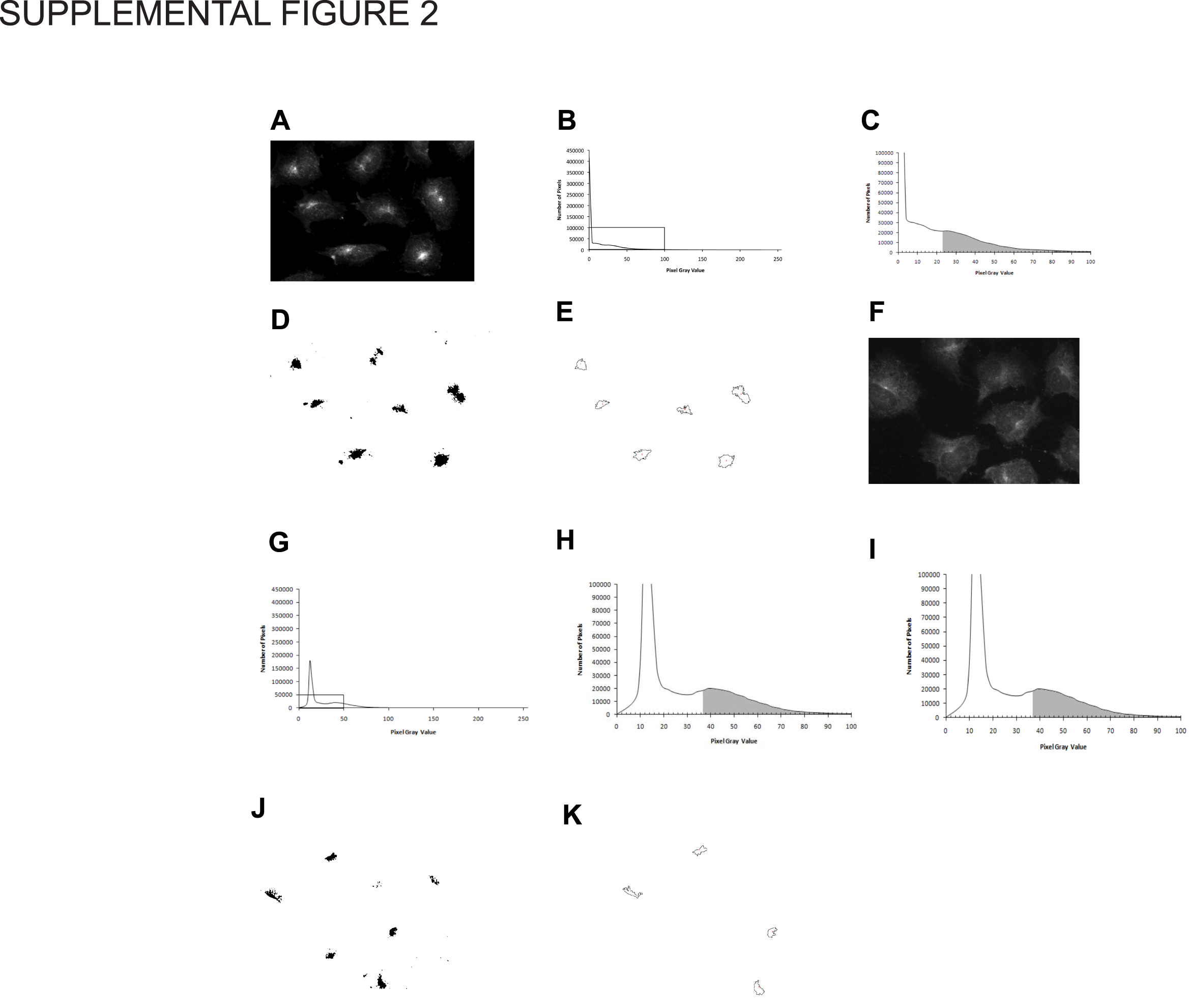

Supplement: Figure S2 — Quantification of staining using Image J. A) Representative image of H4-APP cells for analysis. The cells have been stained for LDLR. B) Histogram generated from ImageJ based on the image shown in panel A. Pixel gray value is the color of the pixel on a scale of 0 to 255, with 0 being absolute black and 255 being absolute white. C) Magnification of the histogram in panel B. In this representative histogram, the mean pixel value is 11.801, and the standard deviation of the histogram is 23.217. In order to identify the intensely stained perinuclear density, the image was thresholded at 3 standard deviations from the mean. At this threshold, only pixels with a gray value of 81 or greater were identified as positive stain (dark gray region in right tail). Identification of total cellular staining was done with the image thresholded at 0.5 standard deviations from the mean of the image, so all pixels with a value of 21 or greater were identified as positive stain (light gray region and dark gray region). For tubulin staining, staining outside of the density was defined as that falling between 0.5 standard deviations and 3 standard deviations (light gray region only). D) Image showing thresholding of the image shown in panel A at 3 standard deviations. Note that only intense staining is identified at this threshold. E) Further identification of the intense perinuclear density based on the criterion that the region be at least 1000 square pixels in area. F–K) Representative image of H4 cells stained for LDLR, thresholded, and perinuclear densities identified as described in A–E. (0.56 MB TIF) [file pone.0008556.s002.tif]
